# Avoid All the Competitive Ones: Dynamics of Altruistic Behavior, Mediators, and Moderators in an Evacuation Drill

**DOI:** 10.3390/bs16060876

**Published:** 2026-06-01

**Authors:** Soyoung Kim, Minsun Song, Fanhao Nie

**Affiliations:** 1 School of Liberal Arts, Seoul National University of Science and Technology, Seoul 01811, Republic of Korea; soyoung.kim@seoultech.ac.kr; 2 Department of Political Science, Valdosta State University, Valdosta, GA 31698, USA; 3 Department of Sociology, University of Massachusetts Lowell, Lowell, MA 01854, USA; fanhao_nie@uml.edu

**Keywords:** fire-drill evacuation, altruistic behavior in situation, altruistic tendency and intent, excessive and desirable competition, stepwise regression, mediation and moderation effect

## Abstract

This study explores how altruistic tendencies translate into altruistic behavior during evacuation and investigates the dynamic roles of altruistic intent and competitive orientations. A total of 127 adults in a lifelong education program participated in a routine fire-drill evacuation conducted in a naturalistic setting, of whom 124 were retained for the final analyses after data screening and exclusion procedures. Situational altruistic behavior was assessed through a post-drill questionnaire, and the findings should therefore be interpreted as self-reported responses within an exercise-based evacuation context. In stepwise regression analysis, altruistic intent emerged as the strongest predictor of self-reported situational altruistic behavior. While altruistic tendency and desirable competition showed positive associations, excessive competition showed a negative association. Mediation and moderation analyses revealed that altruistic intent mediates the relationship between altruistic tendency and altruistic behavior but does not function as a moderator. In contrast, desirable competition operates as a negative moderator, weakening the influence of altruistic tendency on behavior despite its positive association in the regression analysis, while excessive competition exerts a direct negative effect on altruistic behavior. Although altruistic intent plays a key role in translating altruistic dispositions into helping behavior during evacuation, competitive pressures—whether excessive or efficiency-oriented—can constrain the expression of altruistic responses. More broadly, competition does not simply oppose altruism in evacuation contexts but reshapes how altruistic tendencies are expressed during emergency movement, highlighting the importance of managing competitive dynamics to support cooperative and life-saving behaviors.

## 1. Introduction

While disasters may occur randomly, their outcomes are not entirely arbitrary; they are shaped by human decisions and behaviors in each situation (Gantt & Gantt, 2012; Park & Miller, 2006). However, our understanding of human behavior in disasters remains limited and is often clouded by misconceptions (Savage, 2019). This limitation largely arises from the inherent constraints of disaster research: disasters cannot be planned or replicated. Therefore, the public understanding of disasters often extends beyond scientific evidence and becomes shaped by myth-like narratives.

Disasters and risk events are contexts in which understanding human behavior is especially important for effective response planning. However, these situations are inherently difficult to observe systematically, as behavior unfolds under conditions of urgency and disruption. Consequently, misconceptions have persisted, often sustained by myth-like narratives that portray crises as scenes of widespread panic and selfishness (Gantt & Gantt, 2012). Media representations frequently reinforce such narratives, which over time embed themselves in emergency response policies and planning assumptions (Fahy & Proulx, 2009).

Within this context, evacuation should be understood not simply as physical withdrawal from a threatened area but as a socially embedded process involving rapid judgment, interaction, and coordination under time pressure (Sorensen & Sorensen, 2007). Rather than uniformly producing panic, evacuation contexts confront individuals with consequential decisions that may range from self-protective actions to collaborative and altruistic responses (Savage, 2019). Nevertheless, traditional accounts have assumed that urgency quickly overrides deliberation, framing evacuation behavior as predominantly irrational, competitive, and self-interested (Dynes, 1994; Cocking & Drury, 2005).

Panic and competitive behaviors have long been viewed as inevitable during evacuation, leading to the persistent adoption of response frameworks centered on panic in the design of disaster response models (Dynes, 1994). The panic-oriented model assumes that prioritizing individual survival over assisting others constitutes the most adaptive strategy under extreme conditions, as heightened competition is presumed to dominate and helping behaviors are viewed as inefficient in evacuation contexts (Cirillo & Muntean, 2012; Gantt & Gantt, 2012). This assumption is consistent with long-standing evolutionary interpretations of behavior that emphasize individual survival and self-interest as primary drivers of action (Dawkins, 1976; Trivers, 1971).

This panic-oriented perspective overlooks the crucial role of social tendencies, such as altruism, concession, and social identity, that significantly shape evacuees’ responses during crises (Drury et al., 2013; Ntontis et al., 2018). Whether evacuees adopt collaborative or competitive orientations substantially influences the social dynamics that emerge in emergencies (Drury & Cocking, 2007; Gwynne et al., 2015). In particular, altruism warrants greater consideration in disaster response strategies—not merely as a normative ideal, but as an empirically supported social mechanism that promotes collaboration, enhances safety, and facilitates lifesaving action during evacuations (Cocking & Drury, 2005; Kaniasty & Norris, 2004).

Social dynamics are evident across acute evacuation contexts, including aircraft emergencies and fire situations, where individuals may delay their movement to allow others to exit first, yield priority, or assist those experiencing physical or psychological difficulty (Drury et al., 2009). Evidence from fire drills and real fire incidents suggests that such cooperative behaviors can promote coordinated and orderly evacuation under time pressure (Drury et al., 2019; Ntontis et al., 2018). Altruism, therefore, should not be regarded as an exceptional or purely moral response confined to extreme crises but as a recurrent social mechanism that shapes group movement patterns and warrants systematic consideration in evacuation analysis and modeling.

Although altruistic tendency may shape evacuation-related responses, dispositional orientations alone may not directly translate into self-reported situational altruistic behavior. The present study explores altruistic intent as a potential psychological process linking altruistic tendency to perceived altruistic responses during evacuation.

## 2. Theoretical Considerations

### 2.1. Situational Altruism

Altruistic tendencies are often understood as relatively stable dispositions shaped by personal character and moral values (Eisenberg et al., 2002). However, the behavioral expression of altruism is highly contingent on social context, particularly in collective emergency situations (Drury et al., 2009; Batson, 2011). Although individuals may possess enduring prosocial inclinations, whether and how these tendencies are translated into altruistic intent and subsequent action depends on environmental cues, situational urgency, and prevailing social dynamics (Krueger et al., 2001).

In disaster settings, contextual conditions do not merely trigger behavior; they shape altruistic intent and situational responses, reflecting the dynamic interplay between dispositional traits and environmental influence (Darley & Latané, 1968; Ntontis et al., 2018). From this perspective, disaster policy and social systems can be understood as part of the informational environment that structures expectations and conditions under which altruistic intentions translate into action (Dynes, 2006; Tierney, 2014).

Building on this contextual perspective, Dynes (1994) introduced the concept of situational altruism to describe forms of helping behavior that emerge directly in response to the demands of a specific emergency. In crisis contexts, such altruistic responses facilitate the effective mobilization of human and material resources under conditions of urgency.

Unlike everyday altruistic behavior—which may be influenced by considerations of social recognition, reputation, reciprocal relationships, or anticipated rewards (Simpson & Willer, 2008)—altruism in disaster settings is less likely to be driven by long-term social incentives. Instead, crisis-related altruism is often immediate and action-oriented. It arises under conditions of urgency, where the preservation of human life becomes paramount and altruistic intent is rapidly formed in response to the perceived needs of others. Such immediate altruistic behavior is frequently triggered by empathetic responses and reinforced through shared emotional dynamics within the group, which can lead to collective actions aimed at addressing the crisis effectively (Batson, 2011; Haidt, 2001).

The social identity perspective further explains how altruistic responses can emerge among individuals facing a shared threat. A sense of collective identity may develop rapidly in emergency situations, leading individuals to perceive fellow evacuees as members of a common group and increasing the likelihood of helping behaviors such as yielding, assisting, or coordinating movement (Drury, 2012; Drury et al., 2009; Maki et al., 2019).

Situational empathy may further strengthen these responses, as perceived threat heightens sensitivity to the fear and vulnerability of others (Moussaïd & Trauernicht, 2016). Research on prosocial behavior similarly suggests that helping and collaboration can emerge spontaneously under conditions of social interdependence, even in the absence of long-term incentives (Fehr & Gächter, 2002; Bowles & Gintis, 2011). In this sense, situational altruism reflects not a departure from dispositional tendencies, but their activation under the specific environmental and social dynamics of disaster contexts.

From this perspective, altruistic behavior during evacuation may be understood as emerging through intermediate psychological processes shaped by situational interpretation and urgency. In this sense, dispositional altruistic tendencies may influence reported situational altruistic behavior indirectly through the formation of altruistic intent under evacuation conditions.

### 2.2. Altruistic Tendency and Altruistic Intent Under Evacuation

Evacuation under conditions of time pressure, spatial constraints, uncertainty, and perceived threat to life becomes a critical collective task in which individual actions influence not only personal survival but also the safety and efficiency of group movement (Fruin, 1971; Kuligowski, 2011). In such environments, human behavior is often interpreted through two contrasting tendencies: competitive actions driven by urgency and self-preservation, and altruistic responses expressed through helping, yielding, and coordinated movement (Drury & Cocking, 2007; Helbing et al., 2000; Kuligowski, 2011). While altruistic tendencies are generally understood as relatively stable psychological dispositions rooted in individual values and prosocial orientation, their influence on behavior in disaster situations depends on whether these tendencies are translated into situational intent under conditions of urgency (Penner et al., 1995; Ajzen, 1991).

Rather than representing a momentary reaction, altruistic tendency reflects an enduring psychological orientation shaped by individual values, empathy, and broader prosocial dispositions (Batson, 2011; Eisenberg et al., 2006). In everyday social relationships, prosocial behavior—often defined as helping actions that improve the well-being of others—may arise from a variety of motivations, including self-interest, normative obligations, role identity, and empathic concern (Batson, 2011; Snyder & Omoto, 2008). However, the presence of such tendencies does not necessarily guarantee that altruistic behavior will occur in high-risk situations.

Altruistic intent refers to the immediate willingness to assist others when individuals confront urgent needs and perceived threats to life (Dovidio et al., 2006). Unlike relatively stable altruistic tendencies, altruistic intent reflects a more proximal psychological orientation formed in response to situational cues and perceived emergency conditions. In this sense, altruistic behavior can be understood as emerging from underlying tendencies through the intervening formation of situational intent, which is activated by contextual cues such as perceived danger, the vulnerability of others, and shared emergency circumstances (Ajzen, 1991; Batson, 2011).

Under disaster conditions, individuals may rapidly form altruistic intent in response to the fear or distress of others, particularly when the preservation of human life becomes salient (Drury & Cocking, 2007; Drury et al., 2009). Thus, situationally shaped altruistic intent mediates the expression of altruistic behavior during evacuation, even though altruistic tendencies originate within individuals. This perspective is also consistent with cognitive-affective and trait activation approaches suggesting that dispositional orientations are expressed behaviorally through situationally activated motivational and interpretive processes (Mischel & Shoda, 1995; Tett & Burnett, 2003).

While prior research has provided valuable insights into altruistic behavior in disaster contexts, much of this work has focused on helping activities during recovery and post-disaster phases. Fewer studies have examined how altruistic tendencies influence behavior during the evacuation process (Dynes, 1994; Tierney, 2014; Kuligowski, 2011). Because individuals’ decisions and interactions during emergencies shape collective safety outcomes, it is essential to understand the behavioral mechanisms operating during evacuation (Bakhshian & Martinez-Pastor, 2023; Haghani & Sarvi, 2019). However, we still do not sufficiently understand how relatively stable altruistic tendencies translate into situational altruistic intent during evacuation. Therefore, this study concentrates on evacuation as a crucial moment where stable altruistic tendencies can manifest as altruistic intent.

### 2.3. Desirable and Excessive Competition Under Evacuation

Although altruistic behavior is often observed as the dominant response in disaster situations, competitive tendencies may still emerge under conditions of urgency and constraint. Evacuees remain individuals capable of both altruism and competition, and these tendencies may coexist within the same evacuation context (Fehr & Fischbacher, 2003; Bowles & Gintis, 2011). Competition has long been viewed as a fundamental mechanism associated with human survival and social adaptation (Axelrod & Hamilton, 1981; Nowak, 2006). Under conditions of urgency and scarcity—such as limited exits, time pressure, and perceived threats—individuals may engage in competitive actions in an attempt to secure safe passage or access to resources (Helbing et al., 2000; Moussaïd & Trauernicht, 2016). In evacuation settings, competitive behavior may emerge as a situational response to constraints rather than as a deliberate disregard for others, making its effects on evacuation efficiency complex and context-dependent (Cirillo & Muntean, 2012; Haghani & Sarvi, 2019).

Competition has been examined both as an individual disposition and as a social dynamic shaped by environmental conditions. At the individual level, competitive tendency refers to a psychological orientation characterized by achievement motivation, ambition, and the desire to outperform others. Such tendencies are often associated with higher levels of effort and performance in many social and organizational settings (Kayhan, 2003; Shen et al., 2021; Shleifer, 2004).

From this perspective, moderate levels of individual competitiveness may contribute positively to decisiveness and movement efficiency during evacuation, as competitive motivation can prompt individuals to act quickly and avoid hesitation under time pressure (Helbing et al., 2000; Cirillo & Muntean, 2012). Under these conditions, desirable levels of competition may coexist with altruistic tendencies and even interact with them, enabling individuals to act decisively while still engaging in behaviors such as yielding, assisting others, and coordinating movement within the crowd (Helbing et al., 2000; Moussaïd & Trauernicht, 2016; Haghani & Sarvi, 2019).

Beyond individual traits, competition is also shaped by the broader social environment in which individuals interact. When opportunities or resources are perceived as limited, competitive conditions naturally arise within groups, and competitive environments can influence both attitudes and behaviors, including how individuals evaluate others and coordinate their actions (Balliet et al., 2011). However, the effects of competition are not uniform. While moderate competition may enhance responsiveness and reduce hesitation, excessive competition can generate congestion, physical interference, and reduced cooperation among evacuees, ultimately hindering collective evacuation efficiency (Haghani & Sarvi, 2019; Moussaïd & Trauernicht, 2016). Experimental studies indicate that exposure to competitive conditions may reduce subjective well-being, increase negative emotional responses, and weaken the willingness to cooperate or assist others (Brandts et al., 2009).

These dynamics become particularly significant in evacuation situations, where individuals must make rapid decisions under conditions of uncertainty and perceived threat. In such contexts, heightened competitive tendencies may emerge as individuals prioritize personal survival (Shen et al., 2021). When competition intensifies beyond a moderate level, it can undermine self-regulation and increase impulsive behaviors, potentially reducing cooperation and helping among evacuees (Haghani & Sarvi, 2019; Moussaïd & Trauernicht, 2016). The influence of competition on evacuation outcomes is not inherently positive or negative; rather, its effects depend on the intensity of competition and its interaction with the social dynamics of the group. Competitive orientations may function as contextual conditions that strengthen, weaken, or alter the expression of altruistic tendencies during evacuation (Drury et al., 2009).

Understanding evacuation behavior therefore requires distinguishing between desirable competition and excessive competition. Moderate levels of competition may enhance decisiveness and facilitate movement efficiency, whereas excessive competition can disrupt coordination and weaken altruistic responses among evacuees (Helbing et al., 2000; Haghani & Sarvi, 2019). Examining how different competitive tendencies interact with altruistic responses provides a more balanced perspective on human behavior in emergency evacuation contexts. Evacuation effectiveness is best understood not by considering competition or altruism in isolation, but by examining how these tendencies operate in balance within social groups.

Evacuation behavior is shaped not only by stable dispositional tendencies or situational pressures alone, but by the interaction between individual orientations and situationally activated psychological processes under conditions of urgency and social interdependence (Ajzen, 1991; Mischel & Shoda, 1995; Drury et al., 2009). From this perspective, altruistic tendency may become associated with reported situational altruistic behavior through the formation of altruistic intent, while competitive orientations may strengthen, weaken, or alter how altruistic responses are expressed during evacuation (Tett & Burnett, 2003; Sheeran, 2002).

The present study examines altruistic tendency and altruistic intent alongside two different competitive tendencies in evacuation contexts and explores how these forms of altruism and competition influence situational altruistic behaviors such as yielding, helping, and movement coordination during a fire drill. By investigating the interaction between altruistic and competitive responses, this study provides empirical insights into the dynamics of altruism and competition in shaping evacuation efficiency and safety.

## 3. Methods

### 3.1. Setting and Procedure

The study initially involved 127 individuals who took part in a regular fire-drill evacuation conducted in a six-story building at a lifelong education institution located on the outskirts of Seoul. After outlier screening using Mahalanobis distance and exclusion procedures, 124 participants were included in the final analyses. As the evacuation took place during a routine fire drill rather than in a laboratory setting, the study provided a structured yet naturalistic context for examining how altruistic and competitive tendencies may be expressed during evacuation-like movement. However, because the drill did not involve an immediate or real threat, the observed and self-reported responses should not be interpreted as equivalent to behavior in an actual emergency evacuation. Rather, the findings should be understood as reflecting behavioral tendencies and perceived responses within a simulated evacuation context.

All participants were enrolled in a short-term adult training program, with ages ranging from 27 to 42 years. The gender distribution was relatively balanced, consisting of 65 female (52.4%) and 59 male (47.6%) participants. Participation in the activity occurred outside regular course requirements and was not associated with academic credit or other forms of reward. Ethical safeguards were maintained throughout the study procedure. The fire drill was a scheduled institutional safety activity and was not conducted for research purposes. The questionnaire was administered following the drill without collecting personally identifiable information. All data were analyzed in aggregated form, and no individual participant could be identified from the dataset or reported findings. Because the expression of altruistic behavior can be influenced by social relationships and prior interactions (Simpson & Willer, 2008; Fehr & Fischbacher, 2003), participants were asked in advance whether they were acquainted with one another to assess the levels of social familiarity. Participants who reported close acquaintance were advised not to evacuate together during the drill in order to reduce the immediate influence of pre-existing social familiarity on evacuation movement and helping behavior. This instruction was advisory rather than experimentally controlled and was implemented as part of the evacuation procedure.

The fire drill consisted of three phases: (1) room evacuation and group movement, (2) elevator boarding and descent, and (3) assembly at the designated safety area. To simulate emergency conditions, a sense of urgency was induced through typical fire-related alarm sounds. The entire procedure was supervised by a trained fire-drill professional to ensure safety.

Phase 1. Room evacuation and group movement. Following the activation of the fire alarm, participants exited their rooms and moved into the corridor toward the evacuation route. A total of five rooms were used in the drill, each containing approximately 20–30 participants. During this phase, participants left their rooms through the doorway and joined the flow of evacuees moving toward the elevator area. This stage allowed for the observation of interactions at room exits and along the corridor, where individuals adjusted their movement in response to others.

Phase 2. Elevator boarding and descent. Upon reaching the elevator area, participants attempted to board an elevator under simulated fire-drill conditions. The elevator had a maximum capacity of 30 people and descended three floors after boarding. This phase was designed to represent contemporary evacuation contexts, such as high-rise buildings where technological advancements allow the use of fire service elevators during emergencies (Kuligowski, 2003; Ronchi & Nilsson, 2013). The elevator boarding process created a situation in which individuals already inside the elevator interacted with those attempting to board, which allowed for the observation of social dynamics among evacuees. Under these conditions, both concessionary and competitive behaviors could emerge during the evacuation process (Haghani & Sarvi, 2019; Moussaïd & Trauernicht, 2016).

Phase 3. Assembly. After exiting the elevator, participants proceeded to the designated assembly area, completing the evacuation process.

During the evacuation activity, the drill created situations in which participants could encounter behaviors such as hesitation, waiting for others, competitive pushing, overtaking, holding room or elevator doors open, assisting others in exiting rooms or boarding the elevator, and yielding or stepping aside to allow others to pass or board. The elevator boarding and disembarking process heightened participants’ awareness of both concessionary and competitive interactions. Immediately following the drill, participants completed a questionnaire evaluating the extent to which they perceived and reported altruistic or concessionary behavior in this evacuation exercise.

### 3.2. Measures

This study examined altruistic behavior in evacuation contexts by measuring the participants’ altruistic responses in relation to their altruistic and competitive tendencies. The dependent variable was self-reported situational altruistic behavior during the fire-drill evacuation. This measure reflected the participants’ post-drill perceptions of altruistic or concessionary responses within the evacuation exercise rather than direct observation of behavior under actual emergency conditions. Independent variables included altruistic tendency, altruistic intent, excessive competitive tendency, and desirable competitive tendency, all of which were expected to be associated with perceived situational altruistic behavior.

Self-reported situational altruistic behavior was measured immediately after the fire drill using a 10-item questionnaire adapted from established measures of altruism and prosocial behavior (Batson, 2011; Penner et al., 2005; Eisenberg & Miller, 1987) and contextualized for an evacuation scenario. Participants were asked to report their perceived engagement in helping behaviors during the evacuation exercise involving shared space and time pressure. Therefore, the measure should be understood as a self-reported assessment of perceived situational altruistic behavior rather than an independently observed record of actual behavior. The items captured common forms of interpersonal assistance and concessionary behavior observed in evacuation contexts, including informing nearby individuals of a route to the exit, giving up one’s place in the elevator when capacity is limited, and assisting individuals who have difficulty moving during the evacuation. Cronbach’s α for this scale was 0.881. To ensure face validity, the questionnaire items were reviewed by three experts in disaster management, risk management, and measurement.

Altruistic tendency refers to an individual’s inherent disposition to care for and help others, which can influence the likelihood of altruistic behavior in specific situations (Rushton et al., 1981). This trait was measured using the 20-item Altruistic Personality Scale (APS) developed by Rushton et al. (1981). Cronbach’s α for the scale in this study was 0.845. Altruistic intent reflects an individual’s belief in the value of helping others and the intention to engage in helpful actions (Nickell, 1998). It was assessed using the 20-item Helping Attitude Scale (HAS) developed by Nickell (1998). Cronbach’s α for this scale in the present study was 0.855.

To examine the influence of competitive tendencies on altruistic behavior, competitiveness was divided into two dimensions: excessive competition and desirable competition (Kayhan, 2003; Kilduff et al., 2016). Excessive competitiveness was measured using the Hypercompetitive Attitude Scale, which assesses the extent to which individuals strive to defeat others at all costs (Kayhan, 2003). The scale consists of 17 items rated on a 5-point Likert scale ranging from 1 (lowest) to 5 (highest). Cronbach’s α for this scale was 0.847. Desirable competitiveness was assessed using the Personal Mastery Survey (PMS), which captures competitive motivation oriented toward self-improvement and personal development rather than defeating others (Kayhan, 2003). This measure incorporates elements of goal-oriented and self-development forms of competitiveness. Cronbach’s α for the PMS in this study was 0.755.

To strengthen measurement confidence, multiple sources of validity evidence were considered. The dependent-variable items were adapted from established measures of altruism and prosocial behavior and contextualized to evacuation-related situations such as yielding space, informing others of exit routes, and assisting individuals with movement difficulties. In addition, the questionnaire items were reviewed by three experts in disaster management, risk management, and measurement to assess their content validity and contextual appropriateness. Internal consistency coefficients ranged from 0.755 to 0.881, indicating acceptable reliability for self-reported psychological measures. Because desirable competitiveness showed relatively lower reliability than the other scales, findings involving this variable were interpreted cautiously.

### 3.3. Models and Analysis

Stepwise regression analysis was employed to examine the relative influence of altruistic tendency, altruistic intent, excessive competition, and desirable competition on situational altruistic behavior and exclude variables with minimal explanatory power (Hair et al., 2010). This method facilitates the identification of the relative contribution of each independent variable to the dependent variable (Draper & Smith, 1998). In the present study, the analysis was limited to four theoretically derived predictors specified in the study framework rather than a broad exploratory search across numerous variables. However, because stepwise procedures may increase the risk of Type I errors and model instability, the regression findings were interpreted cautiously and considered together with the subsequent mediation and moderation analyses.

Following the identification of influential variables, mediation and moderation analyses were conducted to further examine the relationships among them. The PROCESS macro developed by Hayes (2018) was used to test the mediating and moderating effects of altruistic intent, excessive competition, and desirable competition on the relationship between altruistic tendency and situational altruistic behavior. PROCESS applies bootstrapping procedures based on ordinary least squares (OLS) regression, enabling the estimation of both direct and indirect effects and providing robust inference for mediation and moderation analyses (Hayes, 2018; Preacher & Hayes, 2008).

**Figure 1:**
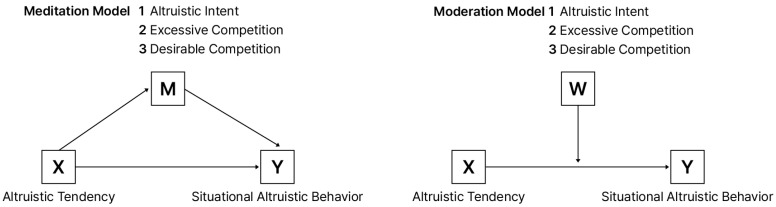
Mediation and moderation models.

Mediation analysis examines the process through which an independent variable becomes associated with, whereas moderation analysis examines how the strength or direction of this relationship varies depending on a moderating variable (Hayes, 2018; Baron & Kenny, 1986; MacKinnon et al., 2007). PROCESS Model 4 was applied to examine whether altruistic intent functioned as a mediating psychological process linking altruistic tendency to reported situational altruistic behavior, and Model 1 was used to examine whether competitive orientations moderated this relationship. PROCESS Model 4 was applied to test the mediation effects and Model 1 to test the moderation effects. This analytical approach was guided by the theoretical premise that altruistic tendency reflects a relatively distal dispositional orientation, whereas altruistic intent represents a more proximal psychological state through which altruistic dispositions may be expressed in evacuation contexts. Competitive orientations were further examined as contextual conditions that may strengthen, weaken, or alter the expression of altruistic responses during evacuation (see Figure 1).

The direct relationship between altruistic tendency and situational altruistic behavior was first tested to confirm the baseline association. Subsequently, altruistic intent, excessive competition, and desirable competition were examined as potential mediators and moderators, resulting in a total of six analytical models.

## 4. Results

### 4.1. Preliminary Analysis

Preliminary data screening was conducted to identify potential multivariate outliers prior to stepwise regression analysis. Mahalanobis distance was examined to detect multivariate outliers that could affect the regression estimates (Tabachnick & Fidell, 2019), resulting in the exclusion of two cases (IDs 45 and 53) from the original sample of 127 individuals, with values of 26.240 (*p* = 0.00008) and 22.977 (*p* = 0.00034), respectively. One additional case (ID 87) was excluded due to early departure from the drill and incomplete questionnaire responses. The total of 124 respondents were retained for the subsequent analyses.

**Figure 2:**
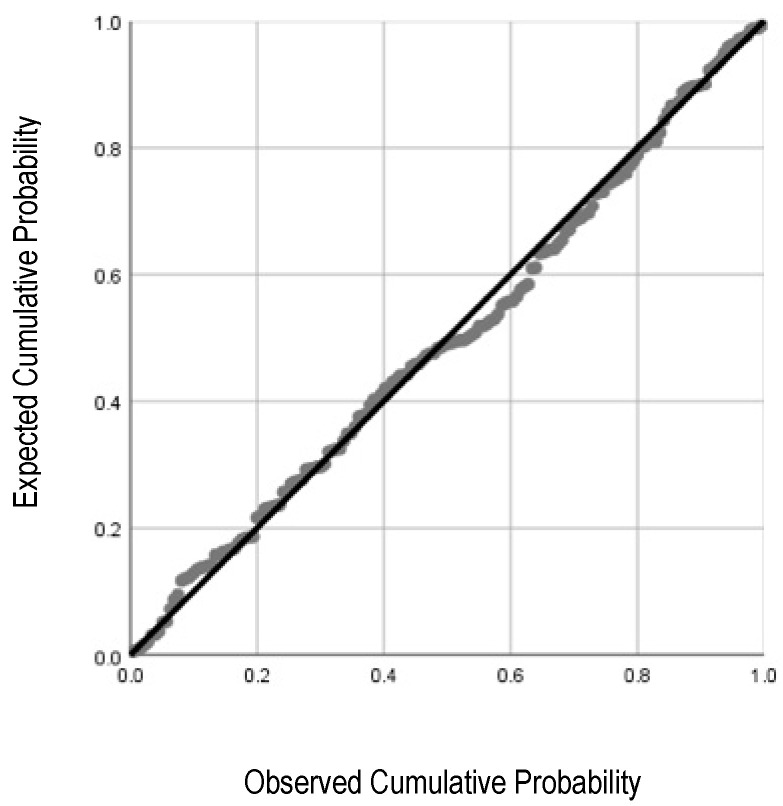
Normative P–P plot on situational altruistic behavior.

Normality of situational altruistic behavior, the dependent variable, was assessed using a P–P plot. As shown in Figure 2, the observed cumulative probabilities (grey points) closely followed the expected cumulative probability reference line (black line), The distribution of the data followed the reference line, indicating no substantial deviation from normality.

The participants had an average score of 3.667 on altruistic intent, which was slightly higher than the average altruistic tendency score of 3.403. The mean score for excessive competitive tendency was 2.775, the lowest among the variables, while the score for desirable competitive tendency was 3.148, higher than that for excessive competitive tendency (see Table 1).

Altruistic tendency demonstrated a significant correlation with both altruistic intent (r = 0.579, *p* < 0.01) and altruistic behavior (r = 0.460, *p* < 0.01). Altruistic intent was significantly correlated with altruistic behavior (r = 0.488, *p* < 0.01) and also showed a significant relationship with desirable competitive tendency (r = 0.266, *p* < 0.01), suggesting a positive connection between altruistic intent and desirable competition. Additionally, desirable competitive tendency had a positive relationship with excessive competition (r = 0.433, *p* < 0.01) and a significant positive relationship with situational altruistic behavior (r = 0.350, *p* < 0.01). Notably, excessive competitive tendency showed negative relationships with altruistic tendency (r = −0.152), altruistic intent (r = −0.139), and altruistic behavior (r = −0.135), though these correlations were not statistically significant.

These correlation patterns also provide preliminary support for the construct validity of the measures. The positive associations among altruistic tendency, altruistic intent, and self-reported situational altruistic behavior were consistent with the expected convergence among prosocial constructs. In contrast, excessive competition showed weak negative associations with altruistic variables, whereas desirable competition demonstrated a different positive pattern. This distinction provides additional support for the conceptual separation between excessive and desirable competition and suggests that the measures captured related but distinguishable constructs.

### 4.2. Stepwise Regression Analysis

Stepwise regression analysis was conducted to examine the influence of altruistic tendency, altruistic intent, excessive competition, and desirable competition on situational altruistic behavior in the fire-drill evacuation. The stepwise procedure produced a model that retained all variables, with none excluded, as detailed below in Table 2. Because the analysis was limited to theoretically specified predictors derived from the study framework, the stepwise procedure was used to examine the relative contribution of each predictor rather than as a broad exploratory variable-selection method. Given the potential risk of Type I errors and model instability associated with stepwise procedures, the findings were interpreted cautiously together with the subsequent mediation and moderation analyses.

In the stepwise regression model for situational altruistic behavior, the variance inflation Factor (VIF) values for all variables ranged between 1 and 3, well below the reference value of 10, indicating no multicollinearity issues (Hair et al., 2010; Tabachnick & Fidell, 2019). The Durbin–Watson statistic was 2.155, within the acceptable range of 1.5 to 2.5, suggesting no autocorrelation problems (Draper & Smith, 1998; Hair et al., 2010).

In the stepwise regression analysis, altruistic intent emerged as the first influential variable on situational altruistic behavior, followed by desirable competition. Altruistic tendency was also significant at the 0.01 level. Excessive competition was a significant variable included in the final validated model and showed a negative effect on situational altruistic behavior.

Altruistic intent and altruistic tendency were positively associated with situational altruistic behavior, whereas excessive competitive tendency showed a negative association. Desirable competition showed a positive association and was examined further in subsequent analyses.

### 4.3. Mediation and Moderation Analysis

Stepwise regression results revealed varying degrees of influence of each variable on situational altruistic behavior, necessitating an explanation of how these differing levels of influence can be integrated into a practical model for evacuation situations. This study hypothesized that altruistic tendency, reflecting a more innate trait, may have a causal relationship with behavior, mediated or moderated by altruistic intent and competitive tendencies. A total of six mediation and moderation models were tested to explore the relationships among altruistic and competitive traits, ultimately leading to the final mediated and moderated model.

**Altruistic Intent.** The mediation analysis conducted using PROCESS Model 4 demonstrated that the model was adequate, with an *R^2^* value of 0.285 and an F-value of 26.576 (*p* < 0.001). The total effect of altruistic tendency on situational altruistic behavior was significant (*t* = 6.002, *p* < 0.001), as was the direct effect (*t* = 2.973, *p* < 0.001). The mediation effect of altruistic intent between altruistic tendency and altruistic behavior was significant, with a 95% confidence interval of 0.080 to 0.337 (See Table 3).

The model for testing the moderating effect (PROCESS Model 1) of altruistic intent between altruistic tendencies and altruistic behavior was validated at a significant level of 0.001, with an R^2^ value of 0.292 and an F-value of 18.142. However, the moderating effect of altruistic intent was not significant, with the value of 0.151 (*p* = 0.276) and the 95% CI lower limit of −0.1222 and upper limit of 0.424. These findings support the role of altruistic intent as a mediator rather than a moderator.

**Excessive Competition.** Regarding the effect of excessive competitive tendency between altruistic tendency and behaviors, the results of conducting mediation analysis with PROCESS Model 4 showed that the mediation model was adequate with the value of R^2^ being 0.216 and the F-value being 18.338 (*p* < 0.001). The total effect of altruistic tendency on altruistic behavior was significant, and the direct effect was also significant (t = 6.002, *p* < 0.001). However, the mediation effect of excessive competition between altruistic tendency and situational altruistic behavior was not significant (95% CI = −0.011, 0.050).

The model for testing the moderating effect of excessive competition between altruistic tendencies and altruistic behavior was validated at a significant level of 0.001 with an R^2^ value of 0.217 and an F-value of 12.216. The moderating effect of excessive competition was also not significant, with the value of −0.054 (*p* = 0.659) and the 95% CI lower limit of −0.295 and upper limit of 0.187. The results indicated that excessive competition functioned as neither a mediator nor a moderator. Furthermore, the effect of altruistic tendency became non-significant when excessive competition was included.

**Desirable Competition.** The results of conducting mediation analysis with PROCESS Model 4 showed that the mediation model was adequate, with the value of R^2^ being 0.290 and the F-value being 27.273 (*p* < 0.001). As the results of testing PROCESS Model 4, the total effect of desirable competition on altruistic behavior was significant (t = 6.004, *p* < 0.001), and the direct effect was also significant (t = 5.620, *p* < 0.001). However, the mediation effect of desirable competition between altruistic tendency and behavior was not significant (95% CI = −0.002, 0.105).

The model to test the moderating effect of desirable competition between altruistic tendencies and situational altruistic behavior was validated at a significant level of 0.001 with an R^2^ value of 0.316 and an F-value of 20.372. Furthermore, the moderating effect of desirable competition was significant at the level of 0.05, with the value of −0.391 (*p* = 0.027) and the 95% CI lower limit of −0.740 and upper limit of −0.043. These results suggest that desirable competition acts as a negative moderator rather than a mediator (see Table 4).

**Figure 3:**
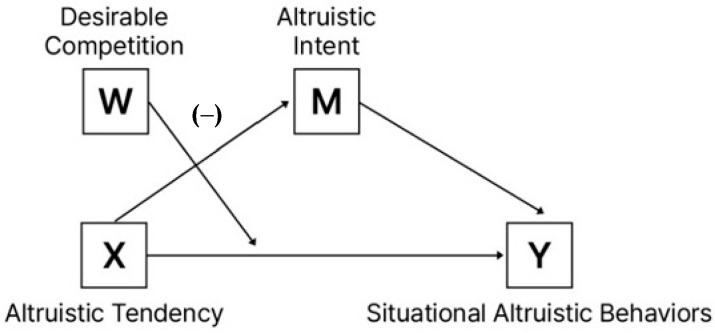
Integrated model based on the results.

**Integrated Model.** Based on these results, a moderated mediation model was constructed using PROCESS Model 5. The model indicates that altruistic tendency directly affects situational altruistic behavior, with altruistic intent serving as a mediator and desirable competition acting as a negative moderator (See Figure 3).

Combining the results of the regression and causal relationship analyses in Table 5, it is evident that altruistic tendency, altruistic intent, and the two forms of competition significantly influence situational altruistic behavior. Altruistic intent mediates the relationship between altruistic tendency and situational altruistic behavior but does not function as a moderator. Excessive competition exerts a direct negative effect on situational altruistic behavior alongside altruistic tendency, whereas desirable competition, although showing a positive association in the regression analysis, acts as a negative moderator in the relationship between altruistic tendency and altruistic behavior.

Based on the stepwise regression results, desirable competition initially appears to promote positive outcomes. However, its role becomes more complex in the relationship between altruistic tendency and altruistic behavior, as it functions as a negative moderator. Because a moderator explains the strength and direction of a relationship, this result suggests that when desirable competition is present, the influence of altruistic tendency on altruistic behavior is weakened. In other words, the competitive element, even when desirable, may shift the focus from altruistic behavior toward achieving competitive goals, thereby reducing the expression of altruism when both traits are present. This finding highlights the complex interplay between altruism and competition in shaping altruistic behavior.

## 5. Discussion

Over the past two decades, social psychological research has increasingly challenged panic-based interpretations of evacuation behavior. Evidence from mass emergencies shows that evacuees often display cooperative and altruistic responses rather than irrational or purely self-interested actions (Cocking & Drury, 2005; Drury et al., 2013; Ntontis et al., 2018). Related studies on collective resilience and prosocial action further support altruism-oriented perspectives on disaster response (Maki et al., 2019).

However, evacuees may also exhibit competitive tendencies, and these behavioral orientations can coexist during emergencies (Savage, 2019). The interaction between altruistic and competitive tendencies may therefore shape behavioral patterns and collective outcomes during evacuation. Understanding how these tendencies operate together is essential for improving evacuation effectiveness and developing practical behavioral guidance that supports both individual and collective safety.

In this study, altruistic and competitive tendencies were explored as two fundamental orientations that may shape evacuation behavior. Rather than assuming that one tendency dominates the other, the study examined how these orientations interact to facilitate or hinder altruistic actions during evacuation, such as yielding passage or assisting others. Importantly, both altruism and competition may emerge to varying degrees in a fire-drill evacuation context. Altruistic orientation may appear as a dispositional altruistic tendency or as situational altruistic intent, whereas competitive orientation may take the form of desirable competition that promotes efficient movement or excessive competition that disrupts coordinated evacuation. Accordingly, this study explored how these forms of altruistic and competitive orientations interact to influence situational altruistic behavior during evacuation.

This study examined altruistic behavior in an evacuation context through a fire-drill activity in which participants displayed a range of behaviors, including hesitation, waiting for others, competitive overtaking, holding doors, assisting others, and yielding passage. Because these behaviors were examined in a routine evacuation drill rather than in a laboratory setting, the context provided an opportunity to examine how altruistic and competitive tendencies may be expressed within a structured evacuation exercise. Nevertheless, the findings should be interpreted as evidence derived from a drill-based and questionnaire-based setting rather than as a direct measure of behavior under actual life-threatening emergency conditions.

Situational altruistic behavior served as the dependent variable, while altruistic tendency, altruistic intent, excessive competition, and desirable competition were examined as independent variables and as potential mediators or moderators. Stepwise regression analysis was conducted to identify the relative influence of these variables on situational altruistic behavior. The baseline relationship between altruistic tendency and situational altruistic behavior was first tested, after which altruistic intent, excessive competition, and desirable competition were examined as potential mediators and moderators through a series of mediation and moderation models.

The preliminary patterns observed in the data suggest that participants generally expressed stronger altruistic intentions and situational altruistic behavior than competitive tendencies during the evacuation drill. Excessive competition appeared relatively limited, whereas desirable competition was more present, indicating that competitive motivations in evacuation contexts do not necessarily emerge in overtly disruptive forms. This pattern suggests that evacuation behavior may be shaped less by direct opposition between altruism and competition than by how different competitive orientations interact with cooperative motivations under urgent conditions.

The findings further indicate that altruistic behavior during evacuation is influenced by both dispositional and situational processes. Altruistic tendency was positively associated with altruistic intent and situational altruistic behavior, while altruistic intent emerged as the strongest predictor of situational altruistic behavior in the regression analysis. This suggests that dispositional altruism is most likely to be expressed behaviorally when accompanied by immediate motivational readiness to help others. In contrast, excessive competitive tendency showed a negative relationship with situational altruistic behavior, implying that strong self-oriented competition may undermine cooperative movement during evacuation. Although desirable competition initially appeared positively associated with altruistic behavior, subsequent analyses suggest that its influence becomes more complex when interacting with altruistic tendencies.

This study explored whether altruistic tendency, reflecting a relatively stable dispositional trait, influences evacuation behavior directly or through more situational motivational processes. Multiple mediation and moderation models were tested to clarify how altruistic and competitive orientations interact in shaping situational altruistic behavior. Altruistic intent plays a mediating role in the relationship between altruistic tendency and situational altruistic behavior. In other words, individuals with stronger altruistic dispositions appear more likely to translate these tendencies into actual helping behaviors when they develop a conscious intention to assist others during the evacuation. In contrast, altruistic intent did not function as a moderator of the relationship between altruistic tendency and behavior. This pattern suggests that altruistic intent operates primarily as a motivational mechanism that channels dispositional altruism into situational action rather than altering the strength of the relationship between tendency and behavior.

Excessive competition did not function as either a mediator or a moderator between altruistic tendency and situational altruistic behavior. This pattern suggests that highly competitive orientations operate as an independent disruptive force during evacuation, encouraging individuals to prioritize personal advancement and effectively “avoid all competitors”, thereby weakening cooperative and altruistic responses.

In contrast, desirable competition showed a different behavioral pattern. Although it did not function as a mediator, it acted as a moderator that weakened the relationship between altruistic tendency and situational altruistic behavior. This finding suggests that even forms of competition perceived as constructive or efficiency-oriented may subtly shift individuals’ focus toward personal performance or rapid progress. As a result, desirable competition may weaken the behavioral expression of altruistic tendencies, suggesting that even socially acceptable competition can constrain altruistic behavior during evacuation.

In conclusion, the findings suggest that within this drill-based and questionnaire-based design, self-reported situational altruistic behavior was associated with dispositional altruism, situational intent, and competitive orientations. Altruistic intent helped explain how altruistic tendencies were linked to reported helping behavior, whereas excessive competition showed a negative association with self-reported situational altruistic behavior. Desirable competition introduced a more complex pattern by weakening the relationship between altruistic tendency and reported situational altruistic behavior. These findings suggest that even efficiency-oriented competition can constrain the expression of altruistic behavior during evacuation, highlighting the importance of carefully managing competitive dynamics in emergency contexts. However, these findings should be interpreted as reflecting reported responses within a structured evacuation exercise rather than as direct evidence of behavior under actual life-threatening conditions.

By examining altruistic intent, altruistic disposition, and different forms of competitive tendency within an evacuation context, this study provides empirical insight into how these orientations shape life-saving behaviors. Both altruism and competition have long been regarded as fundamental forces influencing human survival and social evolution; however, behavioral responses during evacuation are highly context-dependent, as this study suggests. Whereas excessive competition appears to disrupt cooperative behavior, desirable competition demonstrates a more nuanced relationship with altruistic behavior during evacuation.

Interpretation of the present results should take the methodological characteristics of the study design into consideration. Although the mediation model was theoretically specified based on the conceptual distinction between dispositional tendency, situational intent, and perceived behavior, the cross-sectional design permits only the estimation of indirect associations rather than definitive causal mediation (Hayes & Rockwood, 2017; Maxwell & Cole, 2007). Accordingly, the findings should be interpreted cautiously with respect to temporal and causal inference, and future longitudinal or experimental research would help further examine the proposed mediation process.

Furthermore, the evacuation took place during a scheduled fire-drill exercise rather than under conditions involving an actual and immediate threat. Although the drill provided a structured yet naturalistic context for examining evacuation-related movement and interaction, participants were not exposed to the levels of panic, fear, uncertainty, or danger that may arise during a real emergency. Although the measures demonstrated acceptable internal consistency and theoretically meaningful correlation patterns, the study still relied on self-reported questionnaire data. Future research may strengthen measurement validity by incorporating behavioral observation, video-based coding, peer evaluation, or repeated assessment across different evacuation scenarios. The findings should therefore not be interpreted as direct evidence of how individuals would behave under actual life-threatening conditions.

The study also relied on questionnaire-based self-reported responses collected after the evacuation drill. The dependent variable therefore reflected the participants’ reported situational altruistic behavior rather than independently observed emergency behavior. As a result, the findings may be influenced by recall bias, self-perception bias, and socially desirable responding. The study therefore supports conclusions regarding the relationships among altruistic tendency, altruistic intent, competitive orientations, and self-reported situational altruistic behavior within a structured evacuation exercise, but it cannot determine how participants would actually behave during a real emergency situation.

An additional methodological consideration involves the evacuation procedure itself. Participants who reported close acquaintance were advised not to evacuate together in order to reduce the immediate influence of pre-existing social familiarity on evacuation movement and helping behavior. Although this approach was intended to minimize familiarity-related effects and improve internal consistency in the observation of evacuation behavior, it may also have influenced naturally occurring social interactions, potentially affecting the ecological realism and external validity of the evacuation exercise.

Future research may benefit from incorporating more behaviorally grounded observational methods, longitudinal approaches, or simulations involving higher levels of environmental uncertainty in order to further examine how altruistic and competitive tendencies operate under more realistic emergency conditions.

As Auf der Heide (2004) observed, disaster planning is most effective when it is grounded in how people actually behave rather than in assumptions about how they should behave. Designing realistic and effective evacuation strategies therefore requires careful attention to evacuees’ behavioral tendencies and situational motivations. Policies and evacuation models that account for these behavioral dynamics may better support coordinated movement and collective safety during emergencies. Taken together, these findings highlight the importance of minimizing competitive pressures and fostering conditions that allow altruistic tendencies to translate into cooperative action during evacuation.

## Figures and Tables

**Table 1:** Descriptive analysis results.

	Mean	SD	Correlations			
			Altruistic Tendency	Altruistic Intent	Excessive Competition	Desirable Competition
Altruistic Tendency	3.403	0.524				
Altruistic Intent	3.667	0.361	0.579 **			
Excessive Competition	2.775	0.566	−0.152	−0.167		
Desirable Competition	3.148	0.495	0.157	0.266 **	0.433 **	
Situational Altruistic Behavior	3.823	0.559	0.460 **	0.488 **	−0.135	0.350 **

** *p* < 0.01.

**Table 2:** Stepwise regression analysis results.

Model	Variable	*b*	*se*	*t*	*p*	*R* ^2^	Adjusted *R*^2^	VIF
1	(Intercept)	1.651	0.338	4.882	0.000	0.238	0.232	
	Altruistic Intent	0.592	0.092	6.470	0.000			1.000
2	(Intercept)	1.090	0.374	2.916	0.004	0.290	0.279	
	Altruistic Intent	0.516	0.092	5.606	0.000			1.076
	Desirable Competition	0.267	0.086	3.125	0.002			1.076
3	(Intercept)	0.812	0.374	2.174	0.031	0.337	0.322	
	Altruistic Intent	0.329	0.108	3.040	0.003			1.580
	Desirable Competition	0.266	0.083	3.210	0.002			1.076
	Altruistic Tendency	0.284	0.093	3.063	0.003			1.506
4	(Intercept)	1.305	0.414	3.156	0.002	0.368	0.35	
	Altruistic Intent	0.264	0.109	2.426	0.017			1.670
	Desirable Competition	0.388	0.094	4.120	0.000			1.443
	Altruistic Tendency	0.265	0.091	2.906	0.004			1.516
	Excessive Competition	−0.207	0.081	−2.560	0.012			1.386

**Table 3:** Simple mediation analysis: situational altruistic behavior mediated by altruistic intent.

Situational Altruistic Behavior	*b*	*se*	*t*	*p*	LLCI	ULCI
Total effect	0.490	0.081	6.002	0.000	0.329	0.652
Direct effect	0.285	0.095	2.973	0.003	0.095	0.474
Indirect effect		Effect	Boot SE	Boot LLCI	Boot ULCI	
Mediator: Altruistic Intent		0.207	0.065	0.080	0.337	

Note. LLCI = lower limit confidence interval; ULCI = upper limit confidence interval.

**Table 4:** Mediation analysis: Situational altruistic behavior by desirable competitiveness.

Situational Altruistic Behavior	*b*	*se*	*t*	*p*	LLCI	ULCI
Altruistic Tendency	1.771	0.602	2.942	0.003	0.580	2.963
Desirable Competitiveness	1.657	0.606	2.735	0.007	0.458	2.856
Altruistic Tendency × Desirable Competitiveness	−0.391	0.176	−2.224	0.027	−0.740	−0.043
Model	*R*^2^ = 0.316 *F* = 20.376 *p* = 0.000					

Note. LLCI = lower limit confidence interval; ULCI = upper limit confidence interval.

**Table 5:** Mediation and moderation analysis.

Situational Altruistic Behavior	*b*	*se*	*t*	*p*	LLCI	ULCI
Altruistic Tendency	1.610	0.586	2.748	0.006	0.451	2.770
Model	*R*^2^ = 0.335 *F* = 67.723 *p* = 0.000					
Mediator: Altruistic Intent	0.328	0.106	3.086	0.002	0.117	0.538
Indirect Effect	Effect	Boot SE	BootLLCI		Boot ULCI	
	0.167	0.063	0.050		−0.304	
Moderator: Desirable Competitiveness	1.600	0.587	2.723	0.007	0.437	2.762
Interaction Effect	−0.391	0.170	−2.292	0.023	−0.729	−0.053

Note. LLCI = lower limit confidence interval; ULCI = upper limit confidence interval.

## Data Availability

The data presented in this study are available on request from the corresponding author.
